# Young patient with arterial thrombosis and skin changes as the onset manifestations: POEMS syndrome

**DOI:** 10.18632/oncotarget.12570

**Published:** 2016-10-11

**Authors:** Ting-Ting Han, Shuang Zheng, Zeng-Ai Chen, Wei Liu, Yao-Min Hu

**Affiliations:** ^1^ Department of Endocrinology, Renji Hospital, School of Medicine, Shanghai Jiaotong University, Shanghai, China; ^2^ Department of Radiology, Renji Hospital, School of Medicine, Shanghai Jiaotong University, Shanghai, China

**Keywords:** POEMS syndrome, thrombosis, monoclonal protein, young patient, atypical feature

## Abstract

POEMS syndrome is a rare multi-systemic disease characterized by polyneuropathy, organomegaly, endocrinopathy, monoclonal protein and skin changes. Arterial thrombosis is a distinctively unusual feature in patients with POEMS syndrome. We report a 33-year-old man with intermittent amaurosis of left eye and skin changes as the onset manifestations, who was finally confirmed as having POEMS syndrome. Most notably, this was a young man without high risk factors of arterial thrombosis and no monoclonal protein was detected until the repeated measurement later. This case evokes the need to consider the diagnosis of POEMS syndrome for young patients with symptoms of arterial thrombosis but no high risk factors of thrombosis.

## INTRODUCTION

POEMS syndrome, as a rare multi-systemic disease, is characterized by polyneuropathy, organomegaly, endocrinopathy, monoclonal protein and skin changes [1]. There are also several clinical features that not represented in its acronym, such as sclerotic bone lesions, Castleman disease, vascular endothelial growth factor (VEGF) elevation, extravascular volume overload, papilledema, thrombocytosis/polycythemia, clubbing, weight loss, hyperhidrosis, pulmonary hypertension/restrictive lung disease, thrombotic diatheses, diarrhea and low vitamin B12 values [2-4]. All of the above manifestations are thought to be secondary to monoclonal plasma cell-proliferative disorder. Its skin changes are often presented as hyper-pigmentation, hypertrichosis, glomeruloid hemangioma, plethora, acrocyanosis, flushing, and white nails [5]. However, vessel thrombosis is a distinctively unusual feature in patients with POEMS syndrome [6]. In this study, a young man with arterial thrombosis and skin changes as the onset manifestations of POEMS syndrome was reported and similar cases recorded in literature were also reviewed.

## PATIENT DESCRIPTION

A 33-year-old man was admitted to our hospital due to intermittent amaurosis of his left eye that suddenly occurred one month before. He had a one-year history of skin blackening, fatigue, impotence and emaciation. The skin of his face, chest, abdomen, back and limbs, especially that of areolas and armpits, were hyper-pigmented. He also had a history of trauma which resulted in coma for ten days. He had no history of cigarette smoking and his family history was non-specific.

His vital signs were normal, but physical examination showed that he had splenomegaly, scattered warts, lump in the left breast and several lymph nodes palpable in the axillary area. Laboratory tests including blood counts, a biochemistry panel and coagulation function were performed. It was noteworthy for his thrombocytosis (PLT 329×10^9^/L, normal 85-320×10^12^/L), polycythemia (RBC 5.76×10^12^/L, normal 3.68-5.74×10^12^/L), hyperuricemia (UA 476μmol/L, normal 119-428umol/L), dyslipidemia (TG 2.88mmol/L, normal 0.56-1.70mmol/L; HDL-c 0.59mmol/L, normal 0.9-2.0mmol/L), hyperphosphatemia (phosphorous 1.64mmol/L, normal 1.00-1.60mmol/L) and microalbuminuria (UAER 85mg/24h, normal <30mg/24h). Serum autoimmune antibodies (dsDNA, ENA, ANA, ACL, MPO-ANCA and PR3-ANCA) and tumor markers (CA50, AFP, CEA, CA199, CA724, CA211, PSA, fPSA and CA125) were negative. Further adrenal, thyroid, pituitary, gonadal, parathyroid and pancreatic function tests were performed and elevated levels of serum thyroglobulin antibody (TGAb 14.85IU/mL, normal <13.6IU/mL), follicular stimulating hormone (FSH 5.90IU/L, normal 0.8-5.1 IU/L) and prolactin (PRL 23.68ug/L, normal 4.5-12.6 ug/L) were found, but FT3, FT4, TSH, estrogen, progestogen and testosterone levels were normal. ACTH-simulation test showed primary adrenocortical insufficiency (24-hour urinary free cortisol, before/after ACTH simulation 91.70ug/100.06ug) while he had a normal diurnal cortisol rhythm. With the use of 75 g oral glucose tolerance test (OGTT) and insulin releasing test (IRT), a backwardly-shifted peak of glucose-stimulated insulin secretion was indicated (the peaking time of insulin secretion was 120 min) though the glucose tolerance was normal.

**Figure 1:**
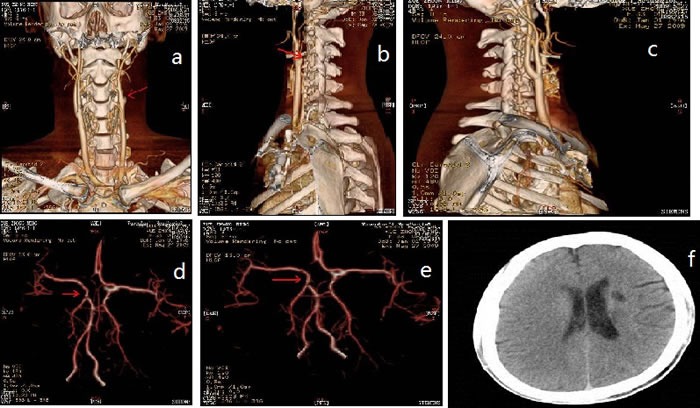
CT-angiography and brain magnetic resonance imaging (MRI) Red arrows: multifocal infarction

**Figure 2:**
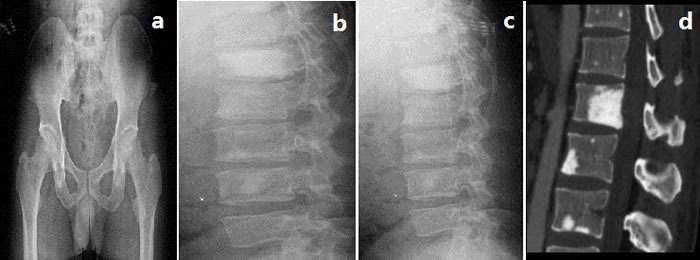
Skeletal radiographic survey and computed tomography (CT)

**Figure 3:**
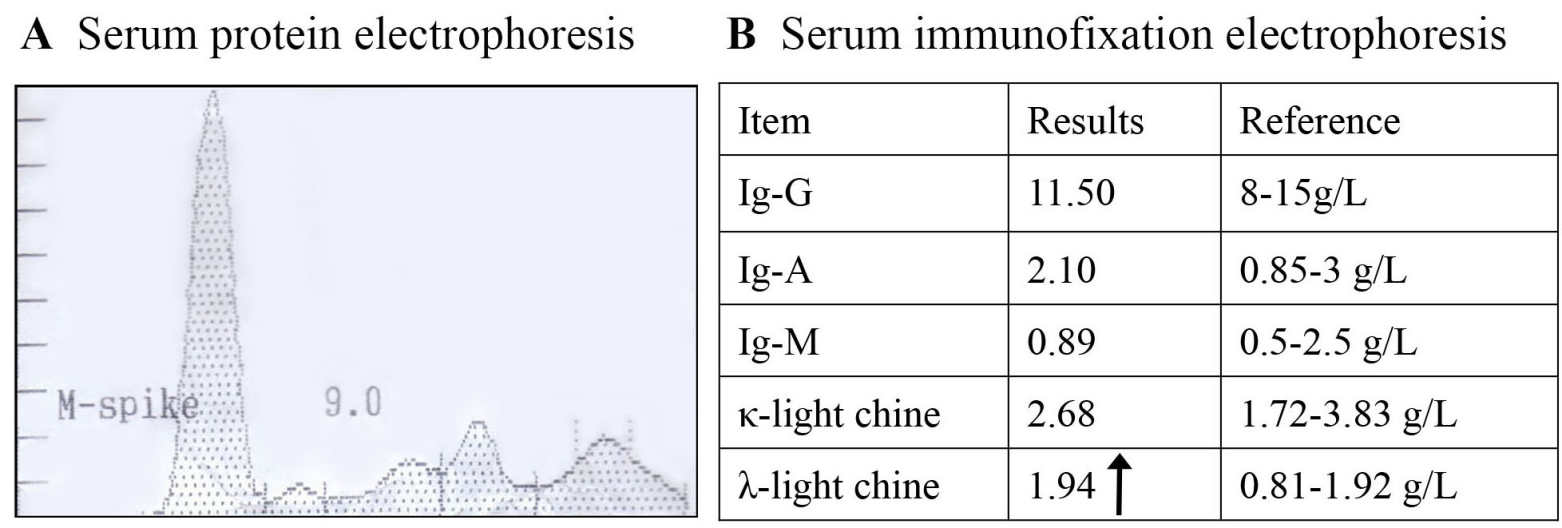
Serum protein and immunofixation electrophoresis revealed monoclonal protein (λ-light chain in the γ region)

Splenomegaly, gynecomastia and enlarged axillary lymph nodes were confirmed by sonography. CT-angiography disclosed occlusion of the left internal carotid artery and disappearance of the left intra-cranial internal carotid artery (Figure 1). Brain magnetic resonance imaging (MRI) showed multifocal infarction beside the left lateral ventricle (Figure 1). A skeletal radiographic survey and computed tomography (CT) scan revealed multifocal sclerotic bone lesions in the thoracolumbar vertebrae and pelvis (Figure 2). On neurologic examination and nerve conduction studies, distal sensorimotor polyneuropathies were noted in his limbs. Bone marrow aspirate and biopsy gave no evidence of plasma-cell dyscrasia, and Bence Jones protein was negative in the urine. Immunofixation electrophoresis test did not detect monoclonal protein in the serum and urine for the first time. One week later, however, we repeated the serum protein and immunofixation electrophoresis in another laboratory and this time the presence of monoclonal protein (λ-light chain in the γ region) was detected (Figure 3).

We diagnosed him with POEMS syndrome complicated by arterial thrombosis. The treatment with anti-coagulants (low molecular heparin, 100 IU/kg, q8h), dexamethasone (37.5mg/d, qw) and melphalan (16mg/d, qw) was started. Platelet count, coagulation indicators (PT, APTT, FIB and TT), and renal and hepatic function were detected regularly. Besides, the condition of patient and other side effects of drugs were monitored. After a period of treatment, his symptom of amaurosis was improved and one month later, he was discharged. Daily drug therapy, healthy lifestyle and regular follow-up visit in hematology department were suggested to the patient.

## DISCUSSION

POEMS syndrome is characterized by the presence of polyneuropathy, organomegaly, endocrinopathy, monoclonal protein and skin changes. However, not all of these clinical manifestations represented in the acronym are mandatory to the diagnosis of POEMS syndrome. Dispenzieri [4] recommended that the diagnosis could be based on having mandatory polyneuropathy and monoclonal plasma cell-proliferative disorder, combined with other major and minor criteria.

Significantly, VEGF elevation, has been listed as one of the major diagnostic criteria. The pathogenesis of POEMS syndrome remains unclear. VEGF can be synthesized and secreted from plasma cells or platelets, and it plays a critical role in edema, microangiopathy/macroangiopathy, and osteosclerotic lesion which induced various clinical features of POEMS syndrome through increasing vascular permeability, angiogenesis and osteoblast differentiation [7]. Except for VEGF, other pro-inflammatory cytokines like interleukin (IL)-1β, IL-6, tumor necrosis factor (TNF)-α have also been implicated in POEMS syndrome disease activity [8].

Our patient fulfilled polyneuropathy, splenomegaly, lymphadenopathy, hypogonadism, adrenocortical insufficiency, impaired insulin release, monoclonal plasma cell-proliferative disorder and skin changes (hyper-pigmentation and angiomas). He also showed multifocal sclerotic bone lesions, thrombocytosis, polycythemia and fatigue. For the diagnosis of POEMS syndrome, this patient met both of the mandatory major criteria, one other major criterion, and four minor criteria.

All patients with POEMS syndrome should have evidence of monoclonal plasma cell-proliferative disorder by definition. However, the amount of monoclonal protein is very small and may be missed on the serum immunofixation electrophoresis. In our case, we did not detect monoclonal protein initially, although it was identified on the second admission. Thus, repeated measurement of monoclonal protein should be essential to confirm the diagnosis of POEMS syndrome.

Sclerotic bone lesion may be a common feature of POEMS syndrome. From the data reported by Dispenzieri et al. [3], 96 of 99 patients (97%) diagnosed as POEMS syndromes in their hospital presented at least one abnormality detected on radiographic bone survey, including sclerotic and/or lytic lesions. Similarly, our patient also showed multifocal sclerotic bone lesions in the thoracolumbar vertebrae and pelvis. The pathogenesis of osteosclerosis in POEMS syndrome is still obscure. One study reported that the lytic lesions of myeloma are seemed to be mediated by the stimulation of osteoclasts by some cytokines, like IL-1β and TNF-α [9]. Thus, it is supposed that the osteosclerotic lesions might be resulted from an unbalance of osteoblast and osteoclast cell activities because of several cytokines. Moreover, platelet-derived growth factors, a kind of cytokines known to stimulate osteoblasts, are thought to be responsible for the diffuse fibrosis and osteosclerosis in agnogenic myeloid metaplasia and other myeloproliferative disease [10, 11], which might also account for the sclerotic bone lesions in POEMS patients.

Although thrombosis is a less-recognized feature in patients with POEMS syndrome, it does not seem to be too rare (Table 1). Lesprit et al. [6] reported that 4 out of 20 POEMS patients had acute arterial obliteration secondary to thrombosis of atheromatous lesions. Moreover, 21 thrombotic events (10 venous, 11 arterial) in 99 POEMS patients of the Mayo Clinic series were also reported [3]. They usually presented as bowel ischemia, limb ischemia, myocardial infarction, stroke and Buddi-Chiari syndrome. In addition, Lee et al. [12] reported a POEMS patient complicated by extensive arterial thromboses who had a fatal outcome within three years. Witoonpanich et al. [13] reported a 16 year-old POEMS patient with thrombosis in left transverse sinus, which resulted in visual failure gradually. Our patient was a young man without high risk factors of arterial thrombosis. However, he had intermittent amaurosis of his left eye as the onset symptom, and image studies demonstrated occlusion of the left internal carotid artery and disappearance of the left intra-cranial internal carotid artery. Arterial thrombosis like our case has been rarely reported. It suggests that thrombosis is a bad prognostic factor, requiring earlier diagnosis and more aggressive treatment.

**Table 1:** Basic characteristics and vessel thrombosis in patients with POEMS syndrome

Number of patients	1[6]	2[6]	3[6]	4[6]	5[12]	6[13]	7[17]
Age	46	53	58	68	41	16	22
Sex	Male	Female	Male	Male	Male	Male	Male
Polyneuropathy	+	+	+	+	+	+	+
Organomegaly	Splenomegaly Lymphadenopathy	Hepatosplenomegaly Lymphadenopathy	Hepatomegaly	Lymphadenopathy	Hepatosplenomegaly Lymphadenopathy	Hepatosplenomegaly Lymphadenopathy	None
Endocrinology	Hypotestosteronemia	Glucose intolerance	Hypothyroidism Hypotestosteronemia	Hypothyroidism Hypotestosteronemia	Hypothyroidism Hypotestosteronemia	Hypothyroidism Hypotestosteronemia	Hypothyroidism Hypotestosteronemia
Monoclonal protein	IgA-λ	IgA-λ	IgA-λ+αIgκ	IgA-λ	IgA-λ	IgA-λ	IgG-λ
Skin changes	+	+	+	+	+	+	+
Sclerotic bone lesions	+	+	+	-	+	+	-
Symptom of thrombosis	Distal ischemia	Monocular blindness	Intestinal colic	Claudication	Dysarthria and left hemiparesis	Visual impairment	Gangrene of left big toe
Site of vessel thrombosis	Iliac	Carotid	Celiac	Iliac and femoral	Bilateral middle cerebral	Left transverse sinus	Femoral, tibial and popliteal
Treatment	Surgery, cyclophosphamide Corticosteroids	Surgery, cyclophosphamide	Aspirin	Surgery, aspirin,	Prednisolone Anticoagulants melphalan	Prednisolone melphalan	Prednisolone melphalan
Outcomes	Died	Died	Died	Died	Died	Died	Alive, distal amputation

The pathogenesis of thrombosis developed in POEMS syndrome is still under discussion. The myeloproliferative and plasma cell disorder, such as thrombocytosis or polycythaemia, may contributed to the development of thrombotic complications in POEMS patients [14-16]. Moreover, Zenone et al.[17] reported a patient with POEMS syndrome who developed arterial thrombosis attributed to essential thrombocythaemia. However, the development of myeloproliferative and plasma cell disorder in the same patient is rare because of the rarity of each of these conditions. A possible explanation for this occurrence is a pluripotent stem cell proliferation with manifestations in two cell lines [18]. Furthermore, pro-thrombotic state and/or vasculopathy in POEMS syndrome may also be caused by increased pro-inflammatory cytokines such as IL-1β, IL-6 and TNF-α. They played important roles in increasing vascular permeability, angiogenesis and osteoblast differentiation [19-21]. Besides, VEGF, released from plasma cells and/or platelets, may induce edema, microangiopathy/macroangiopathy and osteosclerotic lesion, potentially resulting in various features of POEMS syndrome [22].

In conclusion, the patient with arterial thrombosis and skin changes (hyper-pigmentation and angiomas) was diagnosed as POEMS syndrome in our case. Of note, our patient was a young man without high risk factors of arterial thrombosis, and he did not have monoclonal protein detected initially. For patients with arterial thrombosis and skin changes but have no high risk factors of thrombosis, this case evokes the need to consider the diagnosis of POEMS syndrome. Moreover, the repeated measurement of monoclonal protein should be necessary for its diagnosis.
